# Surgical management of urethral obstruction secondary to perineal liposarcoma in a dog: a case report

**DOI:** 10.1186/s12917-024-03956-6

**Published:** 2024-03-23

**Authors:** Natália Korytárová, Beate Bosch, Luise Grace Klass, Pavel Slunsky

**Affiliations:** 1 AniCura Small Animal Specialists Augsburg, Max-Josef-Metzger-Straße 9, 86157 Augsburg, Germany; 2 Anicura Small Animal Specialists Ravensburg, Zuppingerstr. 10/1, 88213 Ravensburg, Germany; 3 Department of Veterinary Medicine, Institute for Parasitology and Tropical Veterinary Medicine, Freie Universitaet Berlin, Berlin, Germany

**Keywords:** Canine liposarcoma, Penile amputation, Preputial urethrostomy

## Abstract

**Background:**

Swelling of the perineal region in male dogs is most commonly caused by a perineal hernia. Clinical signs associated with perineal hernia are constipation, tenesmus or stranguria. This case report documents a rare cause of perineal swelling created by the growth of a malignant tumour leading to urethral obstruction and subsequent stranguria.

**Case presentation:**

An 11-year-old neutered male German Shepherd was presented for swelling in the perineal region and stranguria for three days. Complete blood count and serum biochemistry were unremarkable. Ultrasound revealed a heterogeneous mass in the perineal region. Retrograde urethrography showed a severe narrowing of the urethra caudal to the pelvis. A fine-needle aspirate of the mass was highly suspicious for liposarcoma. Staging was performed by computed tomography (CT) of the thorax and abdomen. Total penile amputation in combination with pubic-ischial pelvic osteotomy, transposition of the remaining urethra through the inguinal canal, V-Y-plasty cranial to the prepuce and preputial urethrostomy were performed to remove the tumour. Histopathology confirmed a well-differentiated liposarcoma with complete histological margins. Six months after the surgery the dog was doing well and there were no signs indicating local tumour recurrence.

**Conclusions:**

Wide surgical excision is generally recommended for soft tissue sarcomas, however this is sometimes not feasible for large tumours. In the case reported here, tumour resection was achieved by a combination of several surgical techniques with a good clinical outcome.

## Background

Liposarcoma is an uncommon malignant tumour of adipose tissue in dogs [1, 2]. Only 0.2 to 0.5% of canine tumours are liposarcomas [2]. Liposarcomas are locally invasive tumours, but metastasize infrequently [1–7]. They are reported most commonly in the axial and appendicular regions, [1, 3, 6, 8–10] but some cases of liposarcomas within the viscera have been reported [3, 4, 6–9, 11]. In rare cases, these tumours can arise from the bone marrow in dogs, (2–3, 5) resulting in extradural spinal liposarcoma [12] or intradural-extramedullar liposarcoma [13]. Lingual liposarcoma [14] and auricular liposarcoma [15] have also been reported.

Recommended treatment of liposarcoma is tumour resection with wide surgical margins. (1, 3–4, 8, 11) Complete resection of the tumour with safety margins can be curative [1, 8]. However, the invasive growth of liposarcomas can make surgical removal challenging [8]. This case report documents a successful surgical removal of a large liposarcoma in the perineal region.

## Case presentation

An 11-year old, 35 kg, neutered male German Shepherd was presented due to swelling of the perineal region and difficulty urinating. The dog showed a weak urine stream and was able to urinate only in small amounts. Hematuria or other changes in the urine were not observed. Medical history revealed that the patient had previously been diagnosed with hypothyroidism and hip dysplasia. Both conditions were under medical treatment at the time of presentation. The dog received levothyroxine (Forthyron, Dechra, Aulendorf, Germany, 10 µg/kg PO q 12 h) and carprofen (Rimadyl, Zoetis, Berlin, Germany, 2 mg/kg PO q 24 h) as long-term medication. During the physical exam, a distended urinary bladder and discomfort on abdominal palpation were noted. A firm, non-painful mass of approximately 7 × 7 cm was detected in the perineal region. During the rectal examination, the mass could be palpated ventral to the colon. Otherwise, the physical exam was unremarkable.

On the day of presentation, blood work was performed. Complete blood count and serum biochemistry panel were within normal limits. Abdominal ultrasound showed moderate dilatation of the intraabdominal portion of the urethra and a distended urinary bladder. Ultrasound of the perineal region revealed a heterogeneous mass. A urinary catheter was inserted to empty the bladder. No crepitation was noted during catheterisation and the placement of the catheter was possible without significant resistance. Urine was macroscopically dark yellow and clear. Urinalysis did not reveal any abnormalities.

**Fig. 1:**
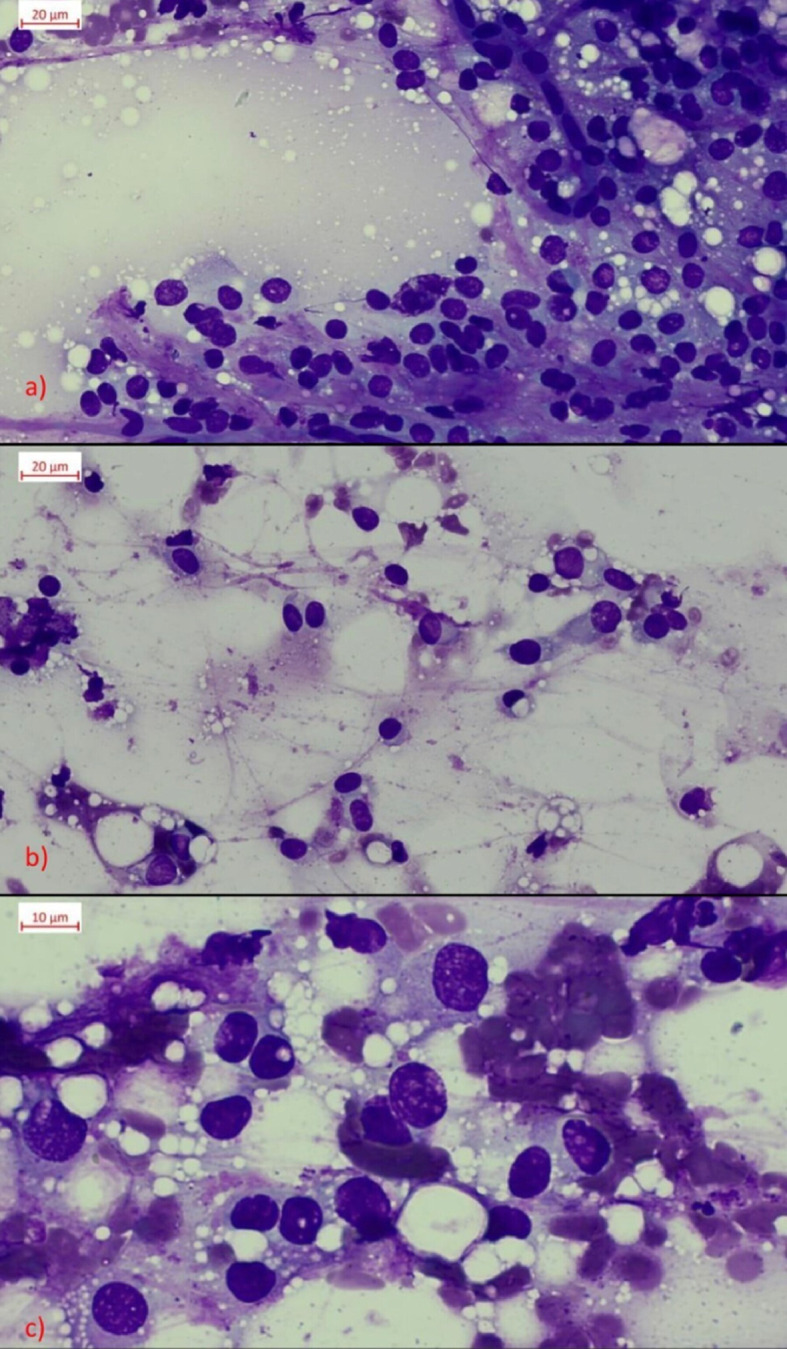
(**a**, **b**, **c**) Cytological finding. Cytologic smears showing multiple pleomorphic fat vacuoles, moderate anisocytosis and anisokaryosis

Ultrasound-guided fine needle aspiration (FNA) of the perineal mass detected rounded, medium-sized cells with a pale, often indistinctly defined cytoplasm. The cell nuclei were round to ovoid with an occasional nucleolus. Many pleomorphic fat vacuoles were present in the cytoplasm (Fig. 1 a,b,c). There was mild to moderate anisocytosis and anisokaryosis (Fig. 1c)). In addition, few well-differentiated mast cells and very few neutrophils were present. The cytological finding was conclusive for liposarcoma.

**Fig. 2:**
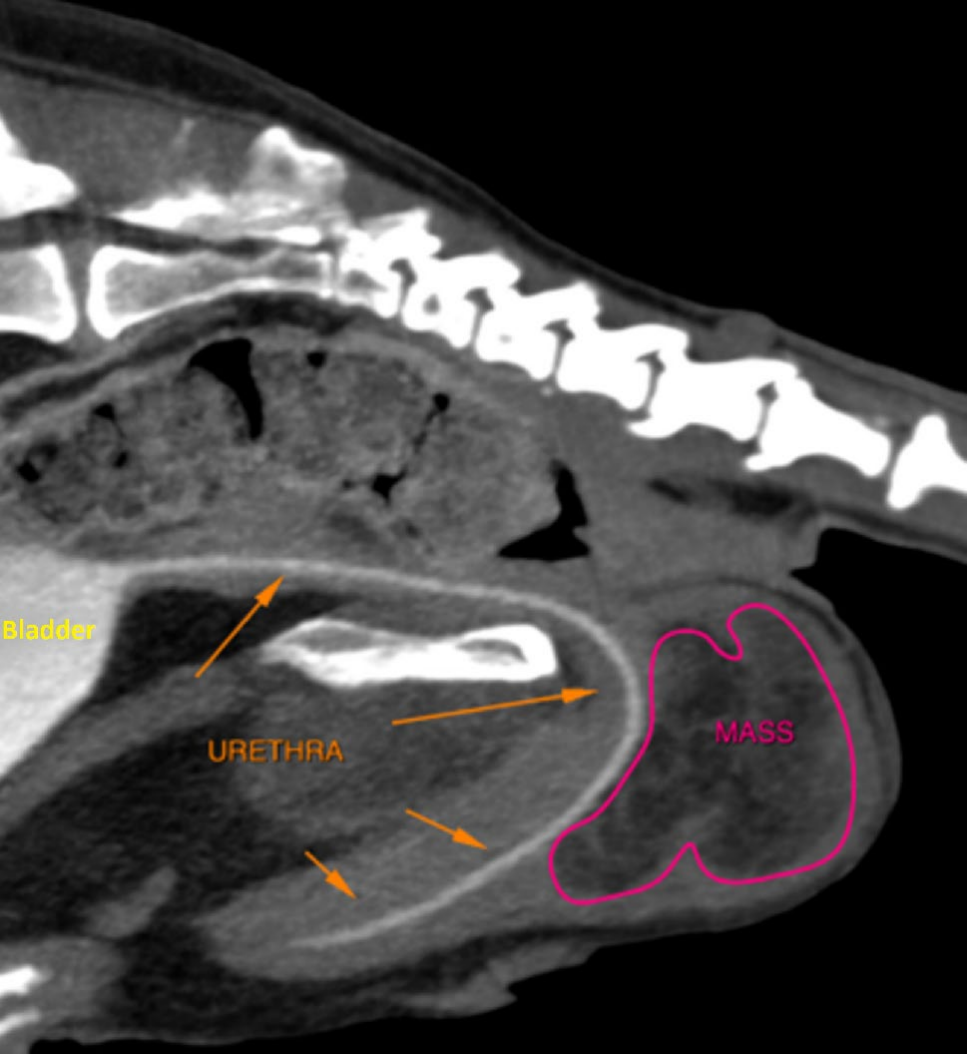
The extent of the tumour on computed tomography. Sagittal view of the pelvic region showing an inhomogeneous mass with heterogeneous fat attenuation and soft tissue streaks caudal to the perineal urethra

The next day, complete evaluation and staging using computed tomography (CT) was performed under general anaesthesia. The CT images revealed a focal mass like lesion within the perineal region caudal to the perineal part of the urethra with secondary mass effect to the urethra (Fig. 2).

The mass was about 45 mm x 65 mm x 47 mm (mediolateral x dorsoventral x craniocaudal) and showed a mild bilobed appearance. It was overall well delineated with a thin soft tissue dense capsule like margin and heterogeneous fat attenuation (Hounsfield Units were native − 40 to -70) with soft tissue streaks. In the late post contrast study, there was none to very mild enhancement of the mass and the capsule. The mass was positioned caudal to the perineal urethra and caudoventral to the rectum. There was a clear distinction between the mass and the rectum due to a thin fat dense region in between. The anatomical origin of the mass could not be determined, but a clear distinction between the mass and the corpus spongiosum could not be identified. The urethra showed continuous contrast filling and overall homogeneous diameter without signs of irregular inner wall layering. Inspection of the regional lymph nodes showed a slight asymmetry between the right and left medial iliac lymph node (13 mm x 12 mm x 25 mm versus 5 mm x 11 mm x 20 mm mediolateral x dorsoventral x craniocaudal), eventhough their size was within the normal range [16]. The remainder of the CT showed unspecific heterogeneous splenomegaly and multifocal degenerative skeletal changes. The chest CT was unremarkable.

**Fig. 3:**
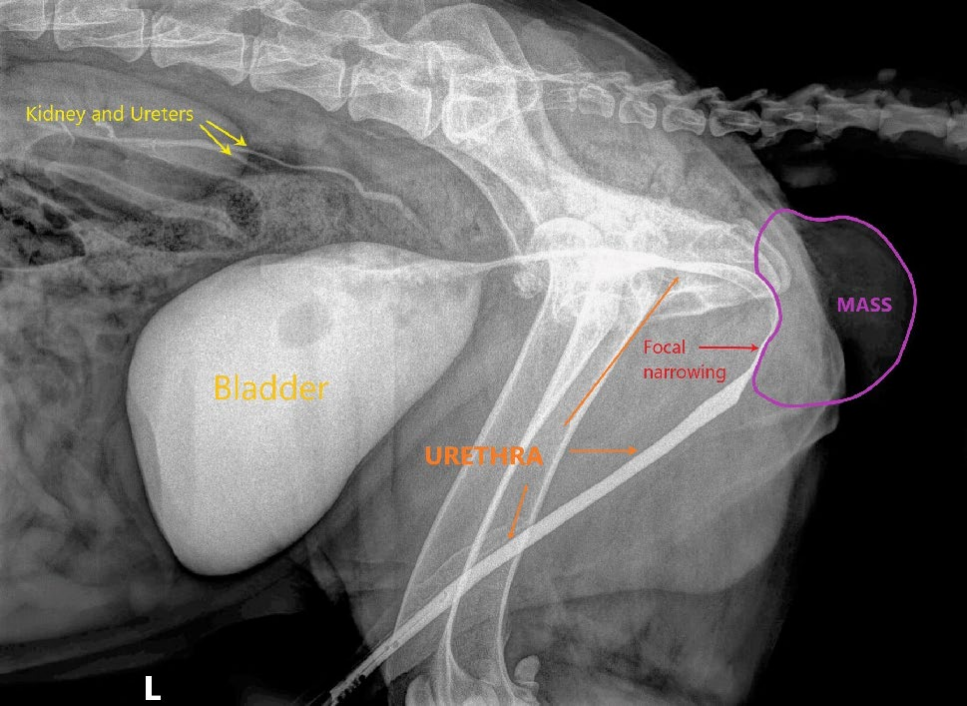
Retrograde urethrography demonstrating severe narrowing of the urethral diameter caudal to the ischial arch

For assessment of the urethra, a retrograde urethrography was performed via a lateral radiograph. This showed a focal narrowing of the perineal urethra secondary to the ill-defined heterogeneous fat attenuating mass like lesion (Fig. 3).

Based on our findings, our suspected diagnosis was perineal liposarcoma associated with intermittent urethral obstruction.

### Surgical treatment

The dog was premedicated with methadone (Comfortan, Dechra, Aulendorf, Germany, 0.4 mg/kg IV) and midazolam (Midazolam, Rotexmedica, Trittau, Germany, 0,2 mg/kg IV). Anaesthesia was induced with propofol (Narcofol, CP-Pharma, Burgdorf, Germany, 4 mg/kg IV to effect) and maintained with isoflurane (Isofluran CP, CP-Pharma, Burgdorf, Germany). After the patient was anaesthetised, epidural anaesthesia using the combination of lidocain (Lidocain-HCl, B Braun, Melsungen, Germany, 4 mg/kg) and morphin (MSI, Mundipharma, Frankfurt am Main, Germany, 0,1 mg/kg) was applied. The dog was placed in dorsal recumbency, and the operating field was clipped and aseptically prepared. A urinary catheter was placed prior surgery. The patient received cephazolin (Cephazolin, Fresenius Kabi, Bad Homburg, Germany, 22 mg/kg IV) as an antimicrobial prophylaxis 30 min before surgery with redosing every 90 min until the end of the surgical procedure.

A ventral midline preputiotomy was performed to expose the penile shaft and preputial cavity. The incision was extended caudally and on both sides of the tumour. The skin above the tumour was resected as wide as possible, to enable tension-free wound closure. Caudally, the mass was well demarcated from the rectum and could be resected without interfering with the external anal sphincter. The penis was isolated up to the ischium. The ischiocavernosus and ischiourethralis muscles were transected. Then, an ischio-pubic pelvic osteotomy was performed after pre-drilling holes into ischium and pubis with a 1.8 mm drill (2 drill holes per bone). The bone flap still attached on one side to the internal obturator muscle was rotated laterally and the intrapelvic urethra was exposed. The urethra, 3 cm cranial to the tumour, was transected and the penis and associated tumour were removed. Surgical instruments were changed after the tumour removal. A ventral caudal midline laparotomy was performed, the transected urethra was separated from the adjacent intrapelvic connective tissue and retracted cranially to the abdominal cavity. The bone flap was repositioned and fixed with 0.8 mm cerclage wire placed through the pre-drilled holes. The adductor muscles were adapted using an absorbable monofilament suture material (MonoPlus 2 − 0, B Braun Surgical S.A., Rubí, Spain). Two stay sutures were placed at the distal end of the transected urethra and the urethra was pulled through the left inguinal canal. The abdominal wall was then closed with a long-lasting absorbable monofilament suture material (MonoPlus 0, B Braun Surgical S.A., Rubí, Spain).

**Fig. 4:**
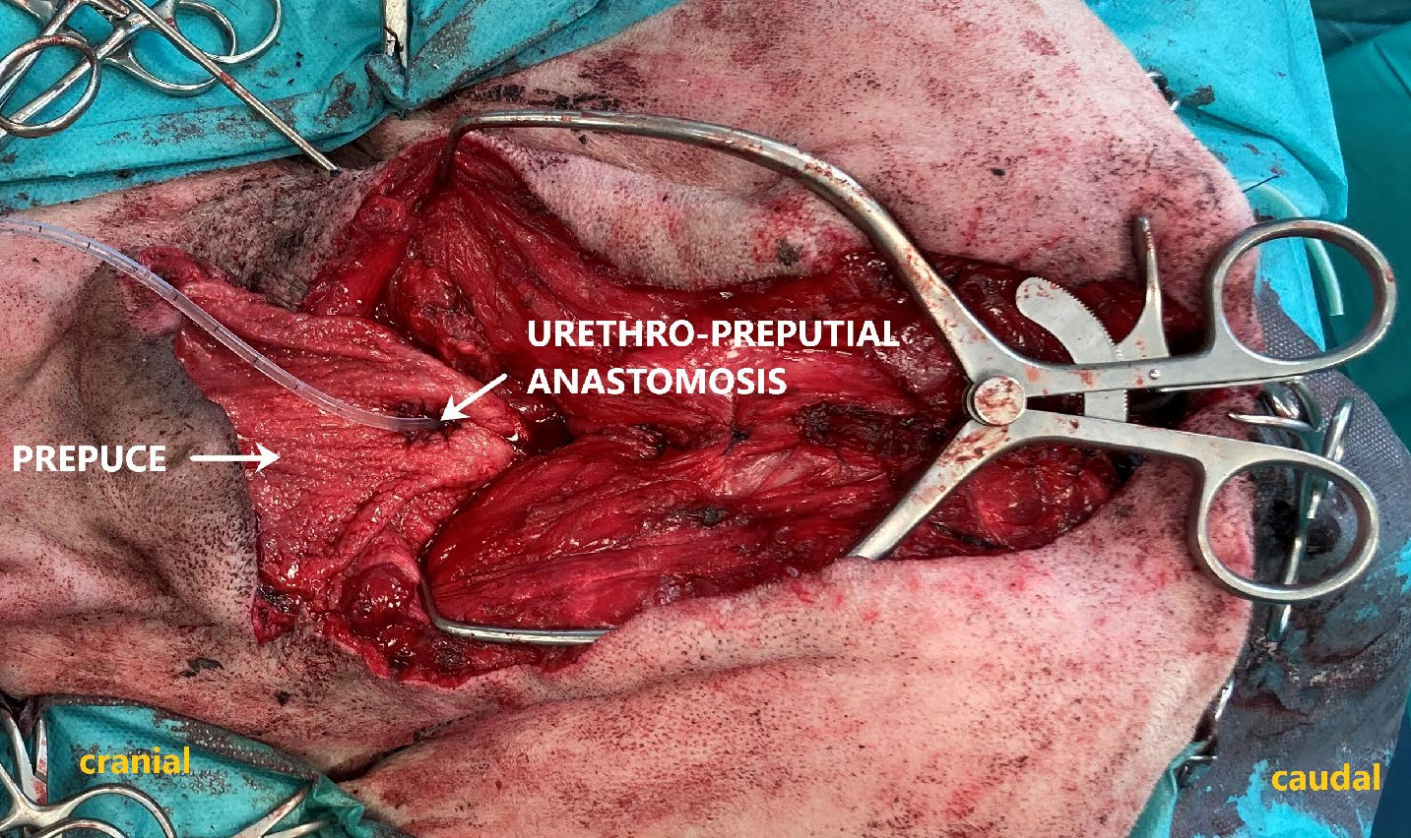
Urethrostomy created within the preputial cavity after tunnelization of the urethra through the inguinal canal

To shift the prepuce caudally, a V-Y-plasty was performed cranial to the prepuce. The caudally shifted prepuce was then sutured on its abdominal side to the abdominal wall with simple interrupted sutures. A stab incision was made in the preputial mucosa, approximately in the caudal quarter of the prepuce, and the urethra was pulled through the preputial incision. After that, the distal part of the urethra was resected, and the urethra was spatulated. An urethro-preputial anastomosis was performed in a simple interrupted pattern using absorbable monofilament suture material (Monocryl 4 − 0, Ethicon, Raritan, USA). An indwelling 8-Fr foley catheter (Smiths Medical ASD, St. Paul, USA) was placed into the urinary bladder (Fig. 4).

**Fig. 5:**
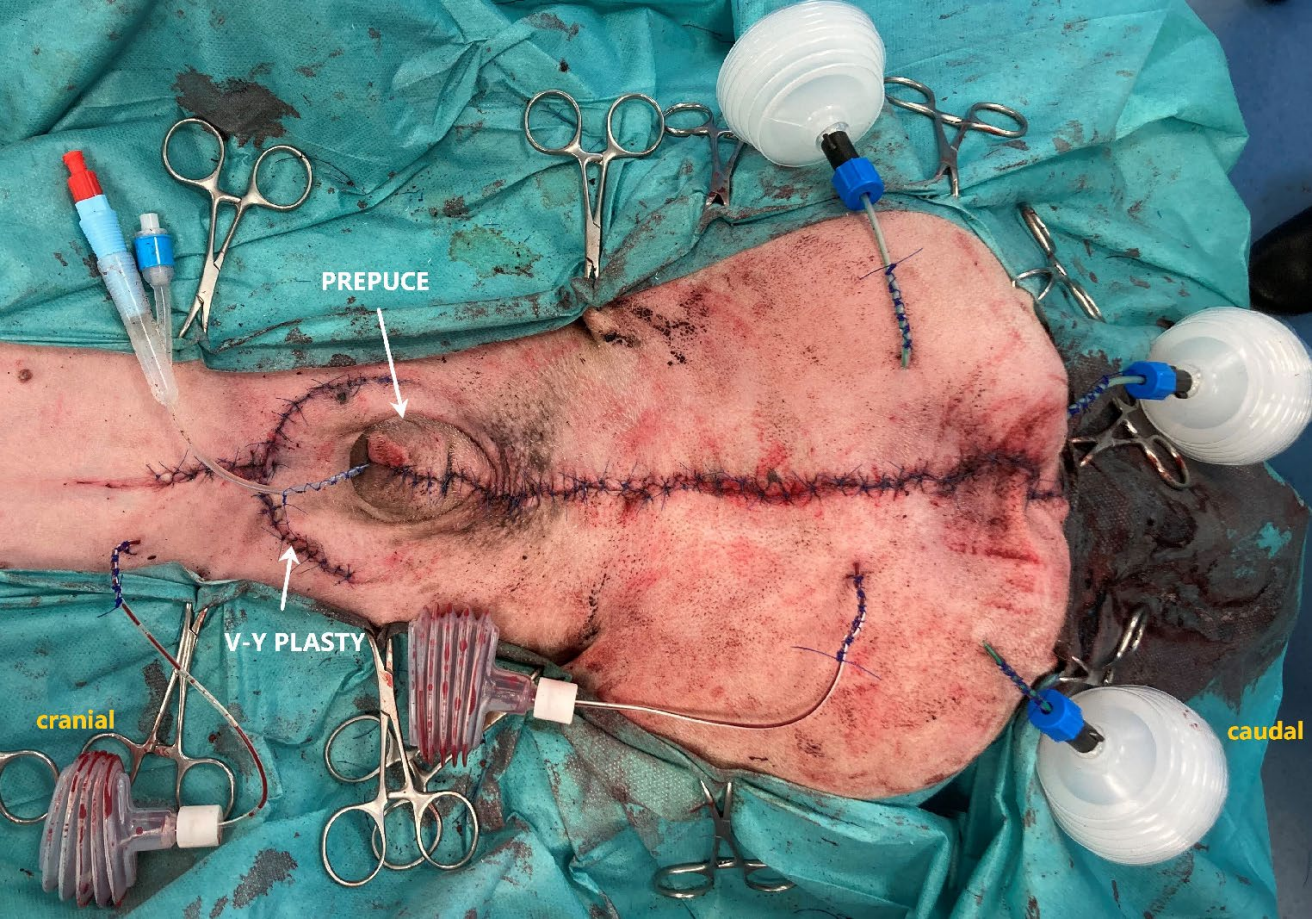
Postoperative image. Wound closure after performing complete penis ablation and resection of the tumour. Cranial to the prepuce, a V-Y-plasty was performed to shift the prepuce caudally. Multiple redon drains were placed into the wound cavity to ensure good wound healing

The preputial mucosa was reconstructed using simple interrupted monofilament absorbable sutures (Monocryl 4 − 0, Ethicon, Raritan, USA). Four redon drains (Eickemeyer, Tuttlingen Germany) were placed into the wound cavity. The V-Y-plasty was drained with one redon drain as well. The subcutis was closed with an absorbable monofilament suture material (Monosyn 3 − 0, B Braun Surgical S.A., Rubí, Spain) and the skin with non-absorbable suture material (Dafilon 3 − 0, B Braun Surgical S.A., Rubí, Spain), (Fig. 5). A control x-ray was taken postoperatively to check the osteotomy reduction and the position of the implants.

Postoperative analgesia was provided as a constant rate infusion (CRI) with fentanyl (Fentadon, Dechra, Aulendorf, Germany, 1mcg/kg/h), lidocain (Lidocain-HCl, B Braun, Melsungen, Germany, 0.6 mg/kg/h) and ketamin (Anesketin, Dechra, Aulendorf, Germany, 0.12 mg/kg/h) for three days. The FLK therapy was gradually reduced before discontinuation of the therapy. The patient was also administered carprofen (Rimadyl, Zoetis, Berlin, Germany, 2 mg/kg PO q 24 h) during the hospital stay. The dog received amoxicillin/clavulanic acid (Clavaseptin, Vetoquinol, Ravensburg, Germany, 12.5 mg/kg PO q 12 h) as postoperative antimicrobial treatment for 7 days. During the hospital stay, the general condition of the patient was good. The drains were removed according to their productivity, when the fluid production fell below 0,2 ml/kg/h. The two cranial drains were removed on day 2 after surgery and the remaining three caudally placed drains were removed on day 4 post-surgery. The foley catheter remained in place for 12 days and the dog was discharged from the hospital on day 13 post-surgery. The dog was continent and able to urinate normally after the catheter was removed.

Histopathology revealed a proliferation of oval to spindle-shaped cells with various cell diameters, high cellularity, cytoplasmic vacuolation, and moderately polymorphic nuclei. On average, less than 1 mitotic figure/10 HPF was observed. Histological margins were complete in all examined sections. Complete histological margins were defined as neoplastic cells > 3 mm from the surgical margin [17, 18]. A well-differentiated liposarcoma was diagnosed.

The first check-up was performed one week after discharge. The dog was doing well, but the owner reported mild wound secretion from the most caudal part of the surgical site. There was an approximately 1 cm long area of wound dehiscence detected on the physical exam. Bacterial culture from the dehiscent surgical site was performed. Local therapy with polyhexanide wound irrigation solution (Prontovet, B Braun, Melsungen, Germany) was started. The wound was cleaned three times a day and the dog had to wear an Elizabethan collar to prevent wound licking. *Escherichia coli* and *Acinetobacter baumanni* were cultured. Systemic antimicrobial treatment with marbofloxacin (Marbofloxacin WDT, Garbsen, Germany, 2 mg/kg PO q 24 h) was started based on the results of the antibiogram. The surgical site healed without further complications. Six months after the surgery, the patient was doing well, urinated normally and did not show any clinical signs indicating local tumour recurrence.

## Discussion

This case report documents a successful surgical removal of a large perineal liposarcoma in a dog using a combination of several surgical techniques.

Tumours arising from adipose tissue can be classified as benign (lipoma, infiltrative lipoma and angiolipoma) and malignant (liposarcoma) [1]. Liposarcomas can be further characterized as well-differentiated, pleomorphic or myxoid [1]. Cytology can often provide a definitive diagnosis, although in some cases the cytological diagnosis may be challenging because of morphological changes in various portions of the same tumour [1, 19]. To differentiate tumours arising from adipose tissue, computed tomography can be helpful [9]. Liposarcomas are characterised as heterogeneous masses with multinodular soft tissue attenuating components, [6, 9] and in some cases associated with regional lymphadenopathy and amorphous mineralization [9]. On CT, liposarcoma typically have medium contrast enhancement, which is not seen in benign tumours arising from adipose tissue [6]. Lipomas are characterised as round to oval-shaped well-marginated fat-attenuating lesions and infiltrative lipomas as homogeneous, fat-attenuating lesions with irregular shape and linear elements, hyperattenuating relative to the surrounding fat [9]. In the case presented here, not all typical CT features described for liposarcoma [6, 9] were represented, however the heterogeneous appearance and fat attenuation of the lesion were in agreement with the suspected diagnosis.

The focal narrowing of the urethra described in the radiograph was not visible on CT likely due to different positioning (lateral versus ventral recumbency) associated with caudal displacement of the mass, creating less mass effect on the urethra. Dilatation of the abdominal urethra and distended urinary bladder detected during the sonographic examination were not visible on the CT image of the contrast study because this examination was performed after the urinary bladder had been partially emptied during catheterization.

The additional CT findings of the asymmetric medial iliac lymph nodes as well as heterogeneous splenomegaly were unspecific. FNA was recommended for further diagnostics, but the owner declined additional investigation.

Recommended treatment of liposarcoma is wide surgical excision. (1, 3–4, 8, 11) Median survival time for patients after wide surgical excision of a liposarcoma is reported to be 1188 days [3]. In case of marginal tumour resection or incisional biopsy, the median survival time decreases to 183–649 days [3]. The intention of the surgery in this case was to remove the tumour completely. In order to increase the chance of complete tumour resection, total penile amputation and resection of the urethra in its intrapelvic portion was performed. However, due to the size and location of the tumour, 3 cm surgical margins, as recommended for soft tissue sarcomas [20], were not achievable laterally. The tumour was resected on both sides as wide as possible for tension-free wound closure. Caudally, the tumour was well demarcated from the rectum and could be resected without interfering with the rectum or the external anal sphincter.

Perineal urethrostomy and transpelvic urethrostomy were considered as alternative surgical methods. However, performing a perineal urethrostomy would in our opinion not be possible without tension on the stoma. In addition, the cosmetic appearance played an important role for the owner, which was the reason why we did not perform a transpelvic urethrostomy and decided to create a new urethral orifice within the prepuce. Urinary diversion via preputial urethrostomy enables the dog to urinate through the preputial orifice, maintains normal anatomy and prevents postoperative urine scalding associated with urethral anastomosis to the adjacent skin [21, 22]. Preputial urethrostomy in dogs was first reported by Bradley, RL (1989), as a surgical treatment of intrapelvic urethral stricture due to trauma [23]. Pavletic and O´Bell (2007) used the prepuce as a site for urethral anastomosis in a dog after subtotal penile amputation due to necrosis of the cranial part of the penis. Preputial urethrostomy was also reported by Katayama et al. (2012), as a surgical treatment in a dog with stricture of the urethral opening after a perineal urethrostomy revision surgery.

Giansetto et al. (2022) described a modified preputial urethrostomy. The main advantage of this approach is its low invasiveness, as penis and prepuce can be preserved [24]. However, all four cases this technique was used on were related to traumatic urethral injury or posttraumatic urethral stenosis [24]. In patients with a malignant urethral or periurethral neoplasia, a partial or complete penile amputation is usually inevitable to ensure sufficient tumour margins. A major disadvantage of the modified preputial urethrostomy is the lack of direct visualization during the urethropreputial anastomosis [24], arguably the most critical part of the procedure, as sutures are placed from the external side of the prepuce. To ensure creating a correct anastomosis, we chose an approach that allowed direct visualization.

Performing a partial penile amputation and an extrapelvic urethral end-to-end anastomosis, as described by Minier et al. (2016), was considered as an alternative approach for our case. However, due to the size of the tumour, there were serious concerns that there would not be enough healthy penile urethra left to create an end-to-end anastomosis after tumour excision. Minier et al. (2016) preserved 3 cm of the urethra caudal to the os penis, which did not appear feasible in our case. Furthermore, urethropreputial anastomosis carries a lower risk of urethral stricture compared to urethral end-to-end anastomosis and, in our opinion, is a less demanding technique.

The necessity of performing a pubic-ischial osteotomy in this case could be arguable. Minier et al. (2016) reported one case of periurethral liposarcoma, in which the urethra was resected caudal to the pelvis and tunnelled cranially into the abdomen without performing a pelvic osteotomy [25]. However, from our point of view, this step allowed us to visualize the entire intrapelvic urethra and its subsequent resection. The reason for avoiding this procedure among surgeons is its invasiveness and possible postoperative complications including inability to ambulate and prolonged recovery time [22]. However there are several reports, in which dogs undergoing pubic-ischial osteotomy were able to ambulate normally immediately after or within the first three days following surgery [22, 26, 27]. In the case presented here, the patient was ambulatory the day of surgery.

The V-Y plasty was initially not planned to be performed. However, it allowed the prepuce to be moved slightly caudally, thereby minimizing the tension on the newly created urethrostomy. Also, the introduction of drains was not originally planned. In oncologic surgery, placement of drains is controversial because of the possible spreading of neoplastic cells. However, significant dead space was created after the tumour was resected, which would predispose the patient to seroma formation. Seroma between the abdominal wall and the prepuce could increase the tension on the anastomosis, thereby increasing the risk of dehiscence of the urethropreputial anastomosis. To ensure drainage of all areas of the wound cavity, several drains were inserted.

The urinary catheter was left in place for 12 days. There are no guidelines determining the length of time the catheter should be left in place and opinions on this topic are conflicting. In some reports, it is recommended to maintain urinary diversion for 7 days [28, 29]. Other research suggests leaving an indwelling catheter for 3–5 days after surgery [30]. There are others who recommend leaving a urinary catheter in place for at least 3 weeks [31, 32]. It is possible that leaving the catheter for 7 days would have been sufficient, but due to the location of the urethrostomy in the prepuce, direct control of the healing stoma was not possible and for this reason the catheter was left in place for longer period of time.

Considering the invasiveness of the surgical resection, placement of a urethral stent was suggested as an alternative. Urethral stenting is an effective and minimally invasive treatment option in patients with urethral obstruction secondary to neoplastic disease [33]. Brückner, M (2022) reported a case of periurethral liposarcoma in a male dog treated with urethral stenting that resolved clinical signs for 11 months [34]. However, since urethral stenting would have been only a palliative solution, the owner declined this option.

## Data Availability

The datasets used and/or analysed during the current study are available from the corresponding author on reasonable request.

## Abbreviations

CT: computed tomography
FNA: fine needle aspiration
HPF: high power field
